# Thermal conductivity of amorphous SiO_2_ thin film: A molecular dynamics study

**DOI:** 10.1038/s41598-018-28925-6

**Published:** 2018-07-12

**Authors:** Wenhui Zhu, Guang Zheng, Sen Cao, Hu He

**Affiliations:** 1 Collge of Mechanical and Electrical Engineering, Central South University, Changsha, 410083 China; 2 State Key Laboratory of High Performance Complex Manufacturing, Central South University, Changsha, 410083 China; 3 Shenzhen StateMicro Electronics Co., Ltd., Shenzhen, 518057 China

## Abstract

Amorphous SiO_2_ (a-SiO_2_) thin films are widely used in integrated circuits (ICs) due to their excellent thermal stability and insulation properties. In this paper, the thermal conductivity of a-SiO_2_ thin film was systematically investigated using non-equilibrium molecular dynamics (NEMD) simulations. In addition to the size effect and the temperature effect for thermal conductivity of a-SiO_2_ thin films, the effect of defects induced thermal conductivity tuning was also examined. It was found that the thermal conductivity of a-SiO_2_ thin films is insensitive to the temperature from −55 °C to 150 °C. Nevertheless, in the range of the thickness in this work, the thermal conductivity of the crystalline SiO_2_ (c-SiO_2_) thin films conforms to the T^−α^ with the exponent range from −0.12 to −0.37, and the thinner films are less sensitive to temperature. Meanwhile, the thermal conductivity of a-SiO_2_ with thickness beyond 4.26 nm has no significant size effect, which is consistent with the experimental results. Compared with c-SiO_2_ thin film, the thermal conductivity of a-SiO_2_ is less sensitive to defects. Particularly, the effect of spherical void defects on the thermal conductivity of a-SiO_2_ is followed by Coherent Potential model, which is helpful for the design of low-K material based porous a-SiO_2_ thin film in microelectronics.

## Introduction

With the continuous miniaturization of integrated circuits (ICs), the characteristic dimension (CD) is shrinking into 10 nm and below^1–4^. Meanwhile, the IC structure has started migrating from 2D to 3D architecture^5^, whose dramatically increased power density brings huge challenges to the performance of resultant microelectronic devices. Owing to excellent thermal stability and insulation property, amorphous SiO_2_ thin films as the dielectric material are widely used in ICs, such as a passivation layer of a semiconductor chip, a charge storage layer in a metal nitride oxide semiconductor (MNOS) memory device, and a gate dielectric layer in an amorphous silicon thin film transistor (TFT). As the CD of ICs decreases continually, the thickness of SiO_2_ thin films shrank into sub-micrometer and even several nanometers that can be comparable to the mean free path (MFP) of hot carriers^6^. Consequently, the conventional Fourier law that describes heat transporting in a way of diffusion could be inapplicable^7,8^. The thermal property of SiO_2_ thin film would be affected by the material size and internal defects as well as external factors such as ambient temperature and mechanical loading. Compared to crystalline SiO_2_ thin films in which heat transfers by phonons with umklapp scattering, heat transport in amorphous SiO_2_ thin films is more complicated for the existence of three regimes of vibrational modes^9,10^. Thus, it is necessary to investigate the heat transport properties of amorphous SiO_2_ thin films.

At present, several works have been conducted to investigate the thermal properties of amorphous SiO_2_ thin films in terms of experiment and simulation. Ratcliffe *et al*. measured the thermal conductivity of crystalline and amorphous SiO_2_ bulk material at different temperatures by steady-state plate methods^11^. They found that the thermal conductivity of crystalline and amorphous SiO_2_ bulk material has exactly the opposite temperature dependence. Lee *et al*. employed the 3-Omega method to measure the thermal conductivity of SiO_2_ thin films with thickness range of 20–300 nm prepared by PECVD^12^. They considered that the observed size effect of thermal conductivity, when the film thickness is less than 50 nm, was attribute to the interface resistance. McGaughey *et al*. studied the temperature dependence of thermal conductivity of amorphous SiO_2_ bulk material using equilibrium molecular dynamics (EMD) method^13,14^. They obtained that the thermal conductivity of amorphous SiO_2_ bulk material was positively correlated with temperature. Using non-equilibrium molecular dynamics (NEMD) method, Huang *et al*. investigated the size effect of thermal conductivity in amorphous SiO_2_ thin films^15,16^. It was found that the thermal conductivity decreased with the thickness of film reducing when film thickness is under 10 nm at 100 K. In addition, Coquil *et al*. performed NEMD simulations to study the correlation between the thermal conductivity of amorphous SiO_2_ thin film and nanopore^17^. Their results were consistent with those predicted by the CP (Coherent Potential) model when porosity was in the range of 10% to 35%. Unfortunately, the potential reason for the consistency between the calculated results and the CP model were not given. In comparison with the published work, the present work systematically investigated the defect-based thermal conductivity tuning in addition to temperature dependence and size effect for thermal conductivity of amorphous SiO_2_ thin films, which would provide useful insights for heat dissipation in IC industry.

## Model and Methods

### Amorphous model of SiO_2_

With respect to the model of amorphous SiO_2_ thin film (amorphous SiO_2_ was simplified as “a- SiO_2_” for short in the following), an atomic model of α-quartz with 49896 atoms (including 33264 silicon atoms and 16632 oxygen atoms) was established, in which the size is 103.173 × 102.115 × 59.55 Å^3^. Then, an NVT ensemble for 10 ps to relax the system at 5 K was implemented. In the following, the system was switched to an NPT ensemble and heated to 5000 K to melt the model. In the end, the temperature of the model was slowly reduced to room temperature (300 K). Thus, the model was fully relaxed (90 ps) at room temperature to obtain a stable structure, and finally the size of the model was stable at 108.665 × 107.550 × 62.619 Å^3^.

**Figure 1:**
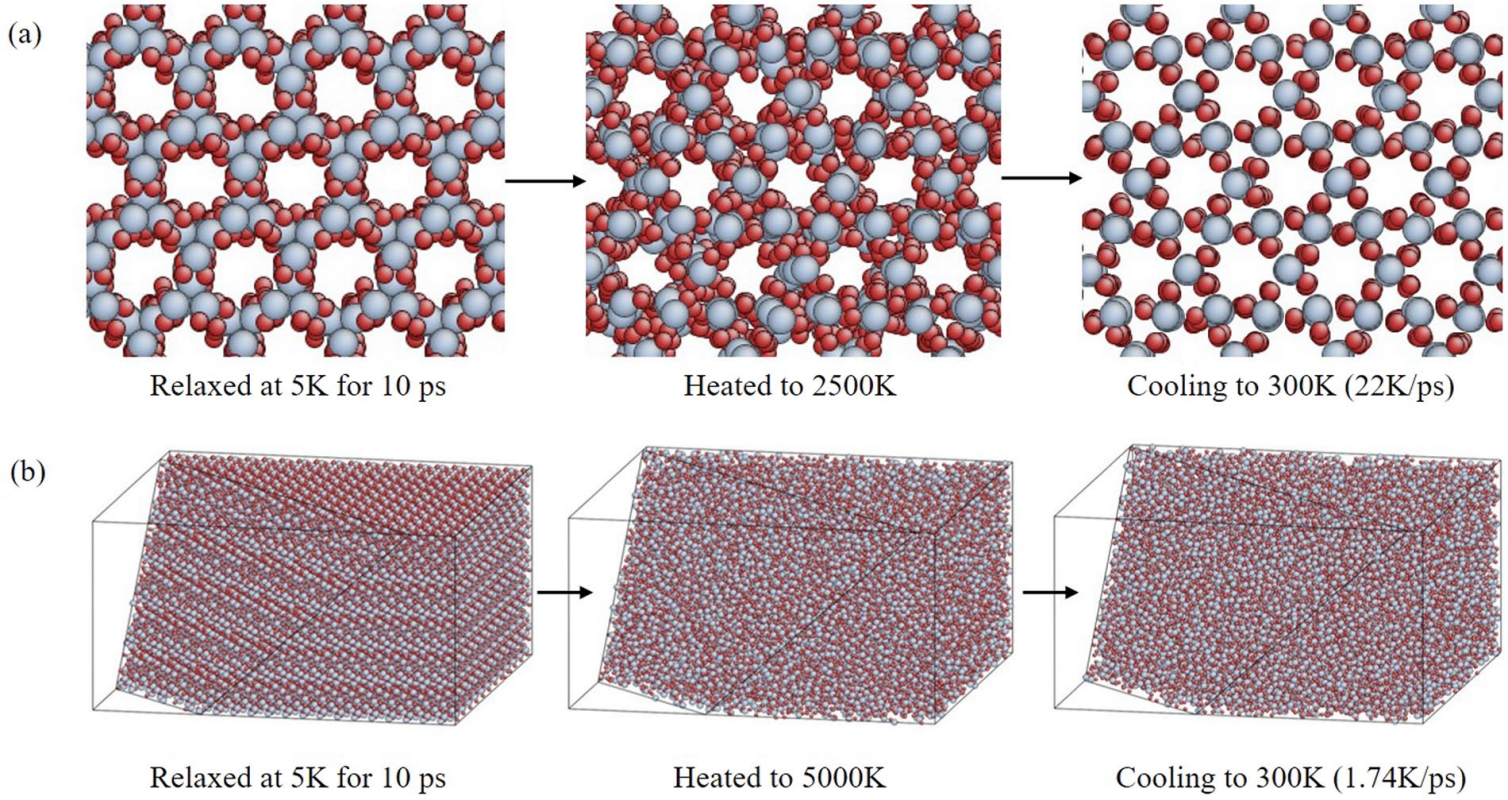
(**a**) Recrystallization with inappropriate parameters in the simulation. (**b**) The amorphization process in this work.

It was found that the heated maximum temperature and the cooling rate are two key parameters during the amorphization process. The heated temperature should make the crystal structure fully changed into disorder state, and the annealing process should be slower, otherwise it will cause the model to recrystallize, as shown in Fig. 1(a). In this work, the cooling rate is 1.74 K/ps. The amorphization process is shown in Fig. 1(b).

**Figure 2:**
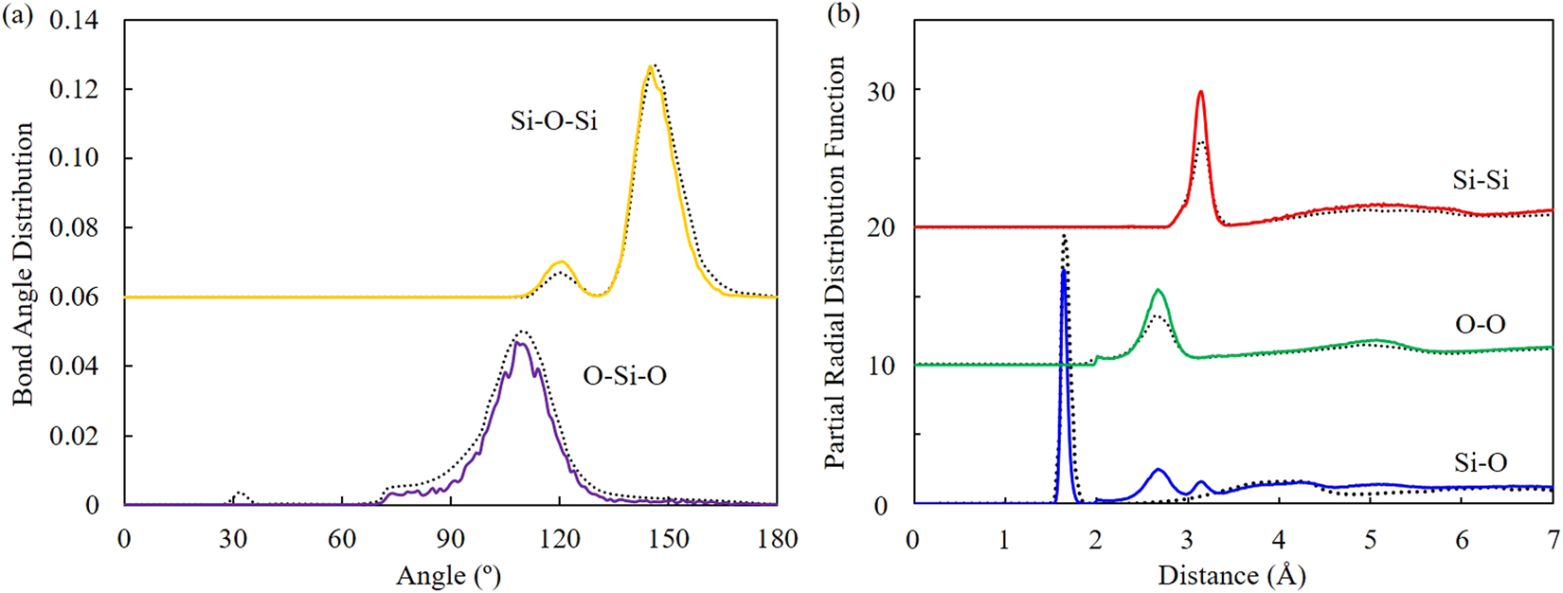
(**a**) The bond angle distribution function for the a-SiO_2_ (solid line, this simulation; dotted line, simulation results from ref.^18^). (**b**) Partial radial distribution function for the a-SiO_2_ (solid line, this simulation; dotted line, simulation results from ref.^18^).

In order to verify the rationality of the model, the bond angle distribution (including O-Si-O bond angle and Si-O-Si bond angle) and the radial distribution function (RDF, including Si-O, Si-Si and O-O) of a-SiO_2_ structural were computed to compared with the literature. The bond angle distribution function and partial radial distribution function for the a-SiO_2_ were plotted in Fig. 2. The solid line is the result of this simulation, and the dotted line is the simulation result from ref.^18^. In Fig. 2(a), the yellow line and the purple line represent the bond angle distribution function of Si-O-Si and O-Si-O, respectively. Obviously, both the highest peak position of Si-O-Si bond angle distribution (144.08°) and the average O-Si-O bond angle distribution (108.72°) are in fairly good agreement with the results of the literature. In Fig. 2(b), the blue line, the green line and the red line represent the radial distribution function of Si-O, O-O and Si-Si, respectively. Overall, the first peak position of the Si-O, O-O and Si-Si are in good agreement with that in the literature. In addition to simulation results, the experimental results were also compared, as shown in Table 1. Compared with results in refs^18,19^, our computed results reveal good consistency, which verify the rationality of the presented model and credibility of selected parameters in this simulation.

**Table 1:** Comparison of structural parameters of the a-SiO_2_ model.

Structural parameters	Our results	Simulation results^18^	Experimental results^19^
O-Si-O bond angle (°)	108.72	109.3	109.4
Si-O-Si bond angle (°)	144.08	146.2	153
Si-O RDF first peak position (Å)	1.65	1.65	1.608
O-O RDF first peak position (Å)	2.67	2.72	2.626
Si-Si RDF first peak position (Å)	3.15	3.16	3.077

### NEMD Method

**Figure 3:**
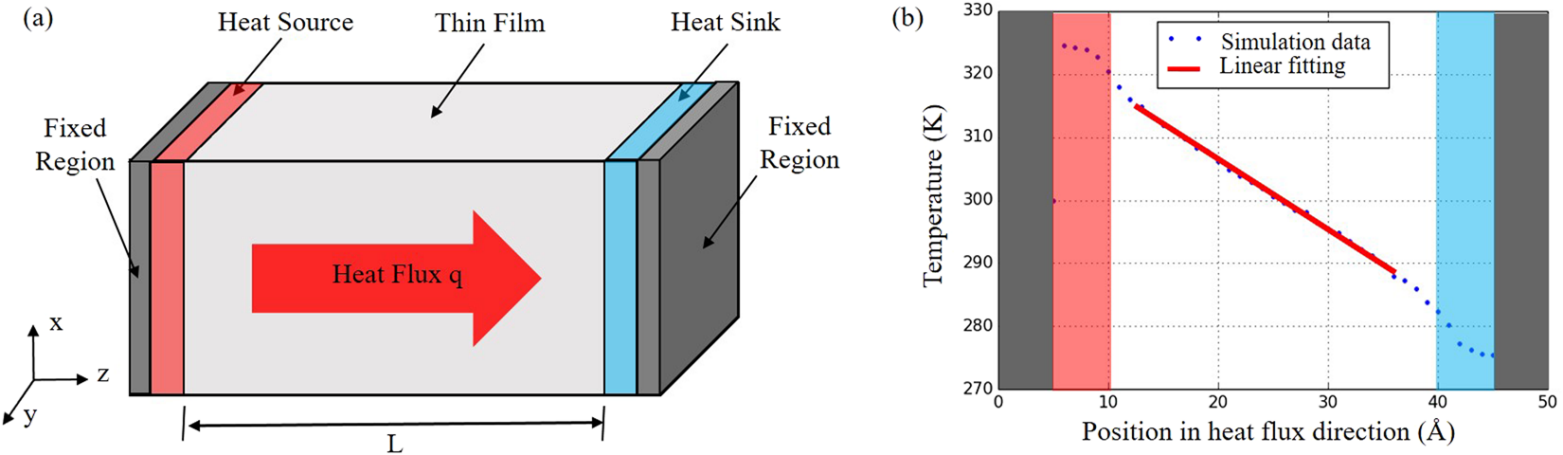
(**a**) A sketch of the simulation domain which consists of the heat transfer region with thickness of L between the heat source and heat sink (about 5 Å in thickness) and the fixed regions (about 5 Å in thickness). And the cross section is about 10.87 × 10.76 nm^2^. (**b**) Typical temperature profile along the heat flux direction.

In our simulations, Tersoff potential function was used to describe atomistic interactions that has been proven to successfully reproduce the structural properties of various SiO_2_ polymorphs in agreement with the ab-initio calculation and experimental results^18,20–22^. The non-equilibrium molecular dynamics (NEMD) method performed on the Large Scale Atomic/Molecular Massively Parallel Simulator (LAMMPS^23^) was used to calculate the thermal conductivity of the amorphous SiO_2_ thin films. The NEMD simulation model, shown in Fig. 3(a), consists of the heat transfer region with thickness of L between the heat source and heat sink (about 5 Å in thickness) and the fixed regions (about 5 Å in thickness). And the cross section of the model is about 10.87 × 10.76 nm^2^. Periodic boundary condition has been applied to the X and Y directions, while periodic and isolated boundary conditions are used in the Z direction (heat flux direction) in accordance with the different steps of the simulation procedure.

The Velocity-Verlet integral algorithm with a timestep of 0.5 fs is applied to solve the Newton’s motion equation. The initial velocity distribution of the system particles is set as Gaussian distribution, and the system is minimized by SD (Steepest Descent) algorithm. Then, the system is equilibrated 100 ps at 300 K in canonical ensemble (NVT, Nosé–Hoover thermostat), and here the boundary condition of the Z direction is periodic boundary condition. Afterward, Langevin thermostat^24^ is used to calibrate the temperature of heat source and heat sink in the NVE ensemble, and here the boundary condition of the Z direction is non-periodic boundary condition. In the following step, temperature gradient is generated by setting the heat source at higher temperature (25 K above the background temperature) and heat sink at lower temperature (25 K below the background temperature). Driven by the temperature difference, thus, the heat flows pass through heat transfer region. Total simulation time during thermal conductivity calculation is 1.25 ns. Energies added into the heat source (E_in_) and subtracted from the heat sink (E_out_) are recorded during thermal conductivity calculation, and then the heat flux (q) is obtained by the following formula,

1 $$q=\frac{{E}_{in}}{A\times \tau }=\frac{{E}_{out}}{A\times \tau }$$

where A is the cross section and τ is simulative time. In this work, the simulation domain is subdivided into 50 bins along the z direction. After reaching steady state, the temperature gradient (∂T/∂z) is obtained by linear fitting of the temperature distribution of each slab, shown in Fig. 3(b). Then thermal conductivity κ is extracted from Fourier’s law,

2 $$\kappa =\frac{q}{\partial T/\partial z}$$

## Results and Discussion

### Temperature dependence of thermal conductivity

**Figure 4:**
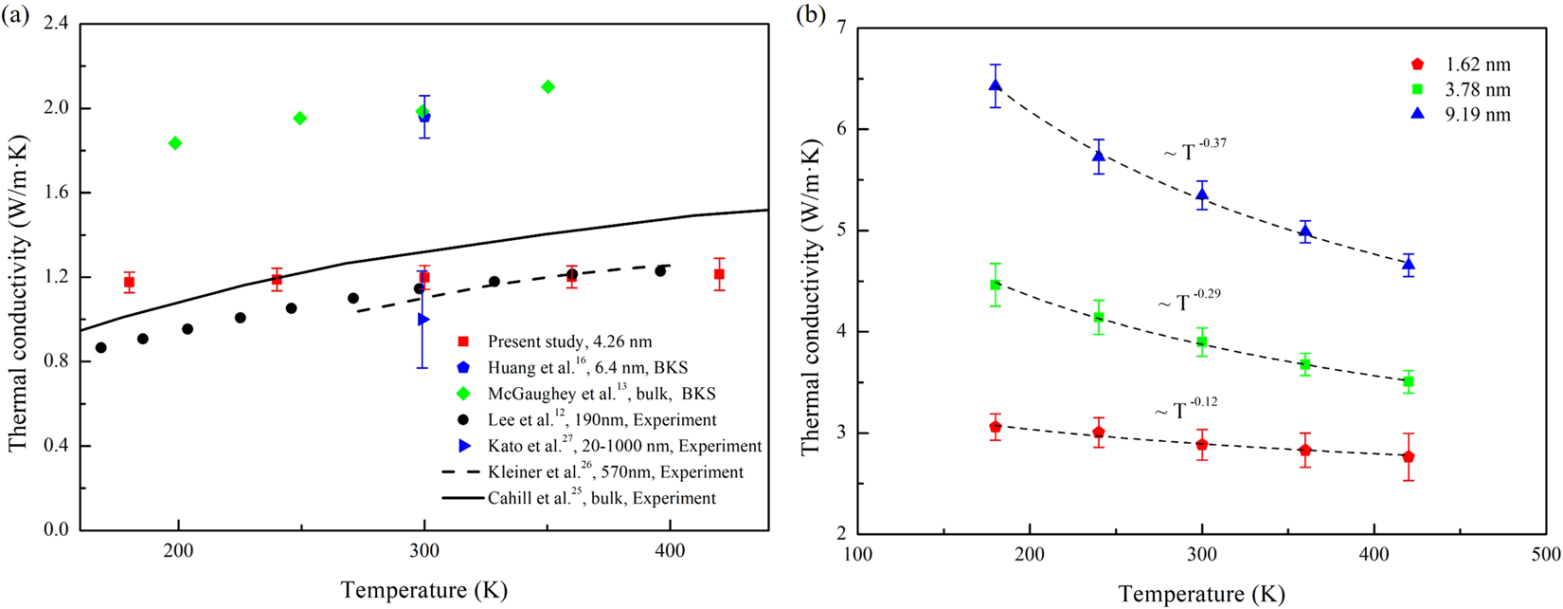
The relationship between thermal conductivity of SiO_2_ thin film and temperature. (**a**) The predicted thermal conductivity of amorphous SiO_2_ thin films based on Tersoff potential with thicknesses of 4.26 nm along with simulation data^13,16^ and experiment data^12,25–27^. (**b**) The predicted thermal conductivity of crystalline SiO_2_ thin films based on Tersoff potential with thickness vary from 1.62–9.19 nm.

Temperature dependence of thermal conductivity of amorphous and crystalline SiO_2_ thin films has been investigated, as shown in Fig. 4. It was found that crystalline thin films and amorphous thin films emerge the opposite temperature dependence. The thermal conductivity of the amorphous SiO_2_ thin films (red squares in Fig. 4(a)) is insensitive to the temperature, while the thermal conductivity of the crystalline SiO_2_ thin films decreases obviously with the increase of temperature.

Figure 4(a) compares the thermal conductivity of amorphous SiO_2_ thin film with thicknesses of 4.26 nm obtained in the present study with simulation results^13,16^ and experimental data^12,25–27^. In Fig. 4(a), the solid line is experimental data of bulk silica reported by Cahill *et al*.^25^, and the green diamonds and blue pentagons are MD simulation data derived from the BKS potential obtained by Mcgaughey *et al*.^13^ and Huang *et al*.^16^, respectively. Obviously, the data obtained from the BKS potential are larger than the thermal conductivity of the bulk silica. The experimental results of a-SiO_2_ thin films obtained by Kleiner *et al*.^26^ and Lee *et al*.^12^ (dashed line and black circles in Fig. 4(a)), show good consistency and exhibit the characteristic of lower than that of bulk silica, and the blue triangle is the thermal conductivity with thickness 20–1000 nm measured by and Kato *et al*.^27^. Compared with the experiment data, our results are close to those beyond 300 K, while slightly larger below 240 K. It’s probably because the Boltzmann energy equalization theorem is strictly effective only when it’s close to or higher than the Debye temperature of the material (Debye temperature of SiO_2_ is 470 K^28^). When the temperature is much lower than the Debye temperature, a large number of phonon modes are not fully excited. In summary, compared with simulation data obtained from the BKS potential, the present results derived from the Tersoff potential are closer to the experimental results, especially when the temperature is greater than 300 K.

Furthermore, the thermal conductivity of the c-SiO_2_ thin film (Here we use “c-” to denote the crystalline structures.) with crystal orientation [001] and the thickness of 1.62–9.19 nm was also investigated to compare the difference between the amorphous film and the crystalline film in this work. As shown in Fig. 4(b), the thermal conductivity of the c-SiO_2_ thin film diminishes evidently with increase of temperature, and follows κ ~ T^−α^ with the exponent range from −0.12 to −0.37. Besides, the smaller the thickness, the smaller the α, which indicates the thinner films are less sensitive to temperature. Compared with the ref.^29^ where the thermal conductivity will show T^−1^ at relatively high temperature, possible reasons for our result T^−α^ are as follows. (1) The specific heat capacity of the material changes with temperature. Since the Debye temperature of SiO_2_ (470 K) is beyond the temperature range we studied, from the Debye model, the specific heat capacity will changed with the temperature. (2) Hu *et al*.^30^ found the exponent may deviate from −1 for short length scale in defect-free system due to the boundary scattering. For the film size of this work has reached the nanoscale, the scattering effect of the thin film boundary on the phonon is also important except for the dominant umklapp scattering of three phonons. The interaction of the two scattering mechanisms determines the effective mean free path of phonon. (3) The high frequency phonon will increase with the rise of temperature, which will lead to the increase of umklapp scattering of three phonons.

In comparison with the temperature dependence of the thermal conductivity of the c-SiO_2_ film and the a-SiO_2_ film in Fig. 4, it can be found that the thermal conductivity of the c-SiO_2_ film is significantly larger than that of the a-SiO_2_, and the temperature dependence is exactly the opposite. The biggest difference between the crystalline film and the amorphous film is that the internal structure of the crystalline thin film is long-range order, but for the amorphous thin film, it is more difficult to describe. The lattice vibration of the c-SiO_2_ film is regular and can be described by the phonon. The heat capacity is also well adhered to the Debye model. Compared to heat transfer by phonons in crystalline materials, heat transfer in amorphous materials is more complicated.

**Figure 5:**
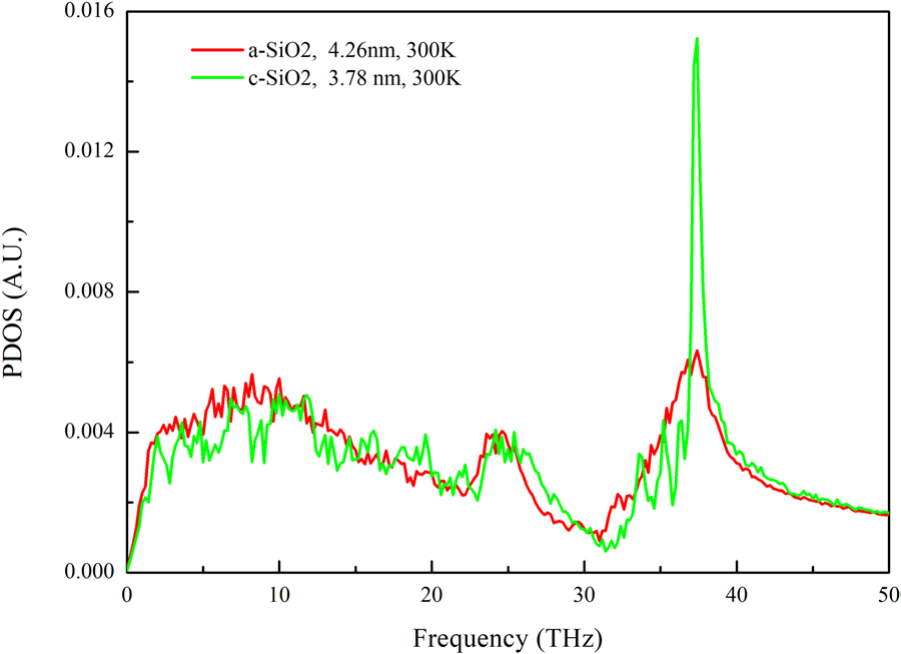
Comparison of the PDOS of c-SiO_2_ thin film and a-SiO_2_ thin film in heat flow direction.

In order to explain the different thermal conductivity between crystalline and amorphous SiO_2_, the phonon density of state (PDOS) of the c-SiO_2_ film and the a-SiO_2_ film were plotted, as shown in Fig. 5. There is no corresponding peak for a-SiO_2_ film at the position of phonon spectra peak for c-SiO_2_ film (37.5 THz). The sharp peak in the Fig. 5 reflects the translational symmetry of lattice^31^, which is not possessed by a-SiO_2_ for no lattice. On the other hand, the reduction in the density of phonon modes will reduce the specific heat of the phonon modes based on the Debye model for the specific heat of acoustic phonons^32^, and thus reduces the thermal conductivity. That is a reason why the thermal conductivity of a-SiO_2_ is lower than that of c-SiO_2_.

### Thickness dependence of thermal conductivity

**Figure 6:**
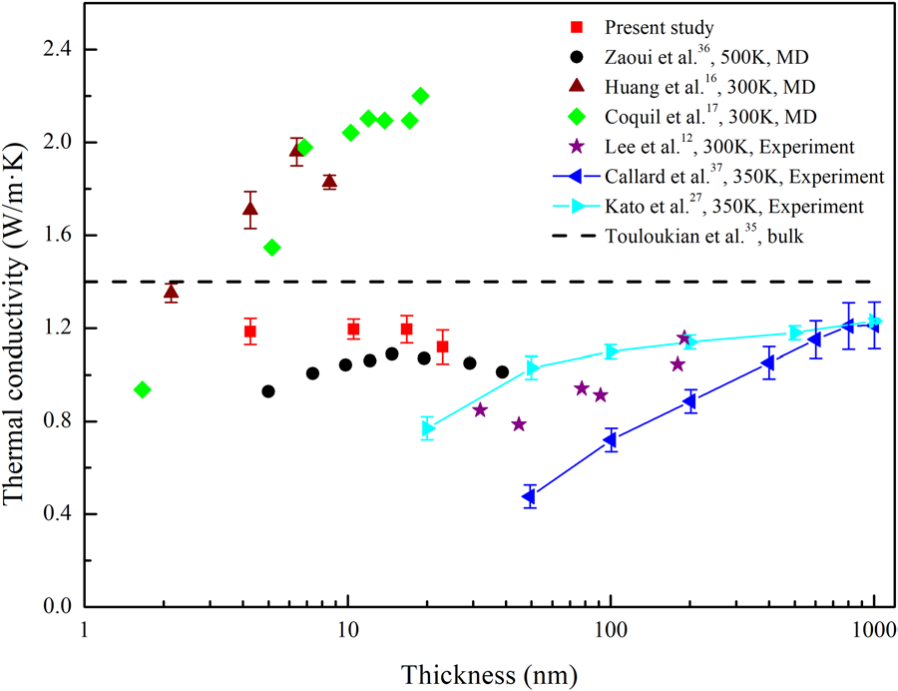
Comparison between the predicted thermal conductivity of a-SiO_2_ thin films at room temperature as a function of film thickness along with previously reported data^12,16,17,27,35–37^.

In this section, the dependence of thermal conductivity of a-SiO_2_ on thin film thickness was investigated at a given temperature (300 K). The cross-sectional area of the system was set to 10.86 × 10.74 nm^2^, and the thin film thickness varied from 4.26 nm to 22.96 nm. As shown in Fig. 6, the thermal conductivity of a-SiO_2_ thin films (red squares) is between 1.1–1.2 W/m·K, and there is no significant size effect.

Strictly speaking, the size effect of the thermal conductivity of the c-SiO_2_ film is interpreted as the boundary scattering of the phonon. However, due to the disorder of the internal structure of the a-SiO_2_, the mean free path of the phonon is extremely short. Kittel^33^ found that the mean free path of the phonon in a-SiO_2_ was only 7 Å (the same order of magnitude as interatomic distance) at room temperature and didn’t vary with temperature, which causes the thermal conductivity of the a-SiO_2_ film related to the heat capacity. Meanwhile, the experimental study of amorphous films by Goodson *et al*.^34^ also showed that the effect of thin film boundary on the scattering of phonon is not important at room temperature.

The results in this work were compared with simulation results and experiment results in the literatures, as described below. In Fig. 6, black circles, wine triangles and green diamonds are the simulation results. Among them, wine triangles and green diamonds are obtained by NEMD method, and black circles is obtained by AEMD method. When the thin film thickness is greater than 4 nm, the thermal conductivity (wine triangles and green diamonds in Fig. 6) predicted by Huang *et al*.^16^ and Coquil *et al*.^17^ based on the BKS potential overestimates the bulk value of 1.40 W/m·K^35^. As previously mentioned by Coquil *et al*., the BKS potential is “somewhat” suitable to simulate amorphous SiO_2_ system. In comparison with BKS potential, the results obtained by using the Teroff potential (black circles and red squares in Fig. 6) are closer to the experiment results. Besides, it was found that our results are close to those obtained by Zaoui *et al*.^36^ when the thickness beyond 10 nm.

Additionally, different experimental values were compared. In Fig. 6, a systematic decrease is observed (cyan triangles, purple stars and blue triangles) in the measured apparent thermal conductivity as a function of film thickness. Lee *et al*.^12^ estimated the most probable explanation is that the apparent thermal conductivity is affected by an additional thermal resistance at the interface between the dielectric layer and the Si substrate and the interface between the heater/thermometer metallization and the dielectric layer. As for cyan triangles and blue triangles in Fig. 6, the experimental values are the effective thermal conductivity, which is related to the film/interface assembly. Both the presence of an interfacial thermal resistance and the intrinsic film thermal conductivity are taken into account. By fitting the results, Callard *et al*.^37^ and Kato *et al*.^27^ obtained the intrinsic thermal conductivity of silica, 1.31 ± 0.11 W/m·K and 1.24 ± 0.04, respectively.

### Void defect dependence of thermal conductivity

**Figure 7:**
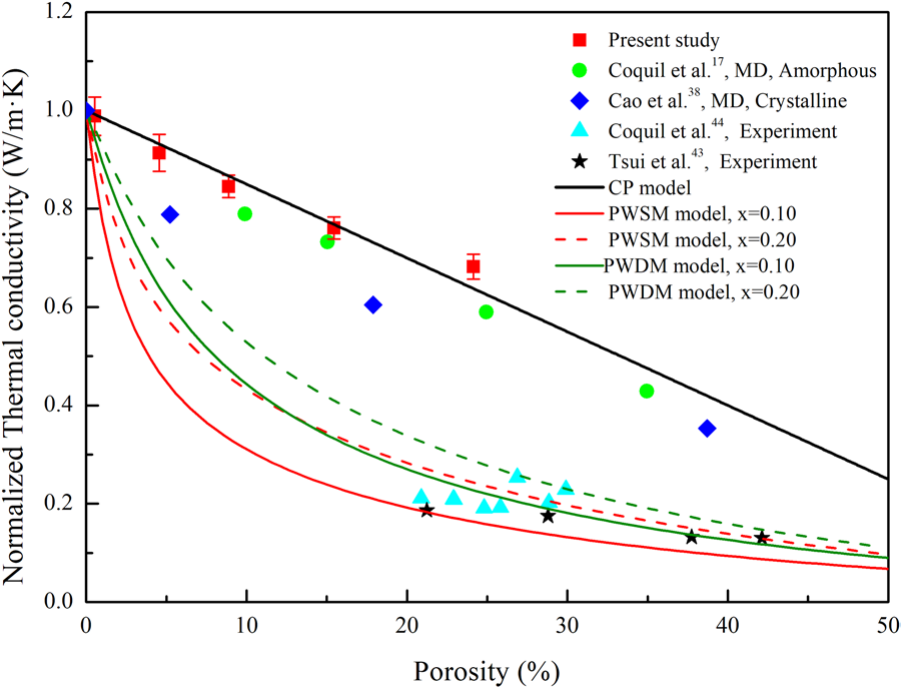
The normalized thermal conductivity as a function of porosity for nanoporous a-SiO_2_ systems of 10.87 × 10.76 × 6.26 nm^3^ along with reported data^17,38,43,44^ and predictions from commonly used effective medium approximations.

The effect of the size of void defect on the thermal conductivity of a-SiO_2_ thin films was further investigated. Defined as the ratio of the number of atoms to be deleted and the total number of atoms in the original model, the void defect is constructed by deleting part of the atom in the model. The thermal conductivity of a-SiO_2_ thin films with different porosity was obtained at 300 K, as shown in Table 2. It was found that the thermal conductivity degrades with increasing porosity, indicating that the presence of voids has an inhibitory effect on the thermal conductivity and the inhibition will be enhanced with the increase of porosity. The effect of the size of void defect on the thermal conductivity of a-SiO_2_ thin films is similar to that of c-SiO_2_ thin films (blue diamonds in Fig. 7)^38^. In order to compare the differences, the thermal conductivity was normalized, as shown in Fig. 7. It was found that the degradation of the thermal conductivity of a-SiO_2_ thin film is relatively smaller, that is, the thermal conductivity of a-SiO_2_ thin film is less sensitive to the size of void defect than c-SiO_2_ film.

**Table 2:** Thermal conductivity of a-SiO_2_ thin films with different porosity.

Porosity (%)	0.539	4.55	8.89	15.44	24.15
κ (W/mK)	1.183 ± 0.04	1.093 ± 0.04	1.009 ± 0.02	0.907 ± 0.02	0.816 ± 0.03

It was obvious that our results (red squares in Fig. 7) were consistent with the CP model among the existing thermal conductivity model of porous media. The CP model was deduced by Landauer^39^ in 1952 to describe the conductivity of the binary metal mixture, which is expressed as follows,

3 $${\sigma }_{{m}}=\frac{1}{{4}}\{({3}{x}_{2}-1){\sigma }_{{2}}+({3}{x}_{1}-1){\sigma }_{{1}}+{[{(({3}{x}_{2}-1){\sigma }_{{2}}+({3}{x}_{1}-1){\sigma }_{{1}})}^{2}+8{\sigma }_{{1}}{\sigma }_{{2}}]}^{{0.5}}\}$$

where *σ*_*m*_ is the conductivity after mixing, *σ*_1_ (*σ*_2_) is the conductivity of component 1 (2), and *x*_1_ (*x*_2_) is the volume ratio of the component 1 (2). Afterwards, Cahill^25^ introduced it to describe the thermal conductivity of the porous material, and succeeded in explaining the reason why the same materials manufactured by different processes have different thermal conductivity. Assuming that the component 2 in equation (3) is void (vacuum) and has thermal conductivity of 0, the relationship between *x*_1_ and porosity p is *x*_1_ = 1 − *p*, so that equation (3) can be simplified to

4 $$\kappa ={\kappa }_{b}(1-1.5p)$$

where *κ* is the thermal conductivity when the porosity is *p*, and *κ*_*b*_ is the thermal conductivity of the bulk material. That is the common expression of the CP model^40,41^.

It’s assumed that the shape of the second phase in the material is spherical when Landauer^39^ derived the equation (3), which is exactly the same as the shape of the defect we considered. That is why our results fit well with the CP model. It was also found that the CP model is applicable in the larger porosity range as well, by comparing the simulation results of Coquil *et al*.^17^ (green circles in Fig. 7).

In addition to the CP model, there are many other models describing the thermal conductivity of porous media, such as the Parallel model, the Serial model, the DF (Dilute Fluid) model, and the DP (Dilute Particle) model. In 1999, Hu *et al*.^42^ proposed the following two models based on the above model: PWSM (Porosity Weighted Simple Medium) model and PWDM (Porosity Weighted Dilute Medium) model, the expressions are shown in equation (5a and 5b).

5a $$\kappa ={\kappa }_{b}\frac{p{\kappa }_{a}+(1-p){\kappa }_{b}}{{\kappa }_{b}}[1-{p}^{x}]+{\kappa }_{a}\frac{{\kappa }_{b}}{p{\kappa }_{b}+(1-p){\kappa }_{a}}{p}^{x}$$

5b $$\kappa ={\kappa }_{b}\frac{2(1-p){\kappa }_{b}+(1-2p){\kappa }_{a}}{(2+p){\kappa }_{b}+(1-p){\kappa }_{a}}[1-{p}^{x}]+{\kappa }_{a}\frac{(3-2p){\kappa }_{b}+2p{\kappa }_{a}}{p{\kappa }_{b}+(3-p){\kappa }_{a}}{p}^{x}$$

where *κ*_*a*_ is the thermal conductivity of air and *x* is the fitting parameter. The PWSM model (PWDM model) is weighted to consider both the Parallel model and the Serial model (DP model and DF model). The weighting factors of the Parallel model (DF model) and the Serial model (DP model) are 1 − *p*^*x*^ and *p*^*x*^, respectively. The fitting parameter *x* which is completely empirical is introduced in order to comprehensively describe the effect of both void shape and size on thermal conductivity. Therefore, the experimental results measured by Tsui *et al*.^43^ (black stars) and Coquil *et al*.^44^ (cyan triangles) were compared, as shown in Fig. 7. When x takes 0.1–0.2, the PWSM model and the PWDM model can match their data well.

Obviously, it was found that the simulation results could not explain the experimental results well. It’s the presence of “necks” connecting the pores in actual amorphous sol–gel mesoporous silica which was ignored in the simulations that could possibly lead to the gap between experimental data and simulation predictions^17^. On the other hand, it was revealed that the porosity only is not sufficient to describe the effect of void defects on thermal conductivity, other factors such as void shape should also be taken into account.

**Figure 8:**
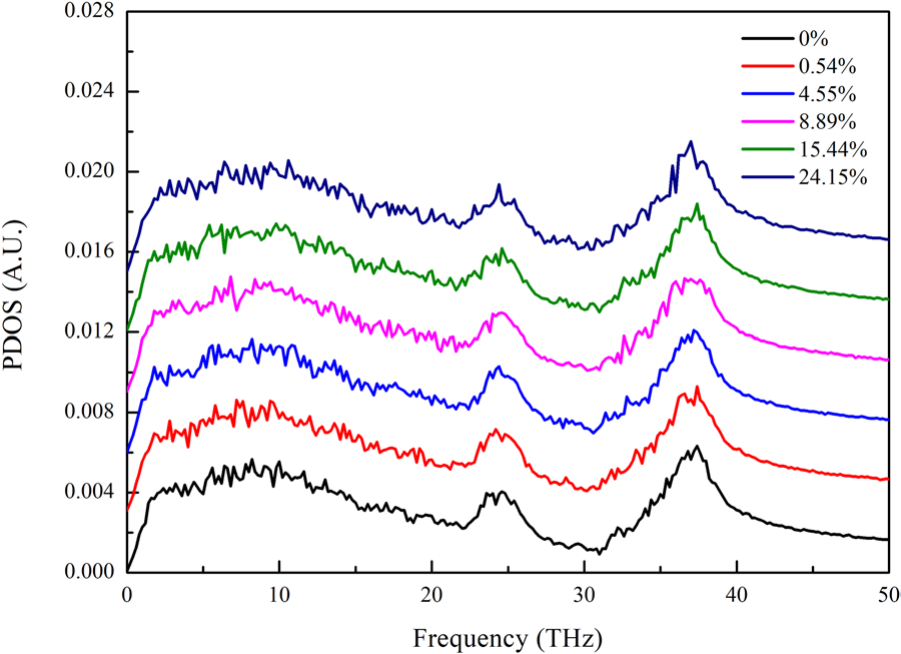
Comparisons of the PDOS among different porosity samples (a shift of 0.003 was adopted at PDOS axis for results clarification).

Figure 8 shows the PDOS of a-SiO_2_ thin films in the heat flow direction with different porosity at 300 K (different colors represent different porosity). In order to show clearly, each curve was translated 0.003 in the vertical axis. There was no obvious difference among the PDOS spectra with different porosity, indicating that the phonon vibrational mode has a poor association with the size of void defect in a-SiO_2_ thin films.

## Conclusions

In this work, the effects of temperature, thickness and void defect on the thermal conductivity of SiO_2_ thin film are investigated by molecular dynamics method. Firstly, the model of amorphous SiO_2_ thin films is obtained by melt-annealing method. And then, the model structures are characterized by calculating the bond angle distribution and the related radial distribution function. The rationality of the model is confirmed by comparing the literature values. Subsequent studies have found that the thermal conductivity of the crystalline and amorphous SiO_2_ thin films in the operating temperature range of the device (180 K to 420 K) has exactly the opposite temperature dependence: In the range of the thickness in this work, the thermal conductivity of crystalline SiO_2_ thin film conforms to the T^−α^ with the exponent range from −0.12 to −0.37, and the thinner films are less sensitive to temperature. While the thermal conductivity of amorphous SiO_2_ thin film increases little with increasing temperature. By calculating the thermal conductivity of amorphous SiO_2_ thin film with different thicknesses and comparing the experimental results and simulation results in the literatures, it was found that the thermal conductivity of amorphous SiO_2_ thin films has no significant size effect when thin film thickness is beyond 4.26 nm. In the study of periodic nanopores, the thermal conductivity of amorphous SiO_2_ thin film was found less sensitive to defect than crystalline SiO_2_ thin film. At the same time, according to the calculated values in the literatures, the effect of spherical porosity on the thermal conductivity of amorphous SiO_2_ thin films can be described by CP model, which is related to the basic assumptions of the model. In addition, it is found that the porosity only does not adequately describe the effect of void defect on thermal conductivity, other factors such as void shape should also be taken into account which is our next research topic.
